# Integrative analysis of physiology, biochemistry and transcriptome reveals the mechanism of leaf size formation in Chinese cabbage (*Brassica rapa* L. ssp. *pekinensis*)

**DOI:** 10.3389/fpls.2023.1183398

**Published:** 2023-04-06

**Authors:** Lixia Wang, Shu Zhang, Ye Zhang, Jingjuan Li, Yihui Zhang, Dandan Zhou, Cheng Li, Lilong He, Huayin Li, Fengde Wang, Jianwei Gao

**Affiliations:** ^1^ Institute of Vegetables, Shandong Academy of Agricultural Sciences, Jinan, China; ^2^ College of Life Science, Huangshan University, Huangshan, China; ^3^ College of Life Sciences, Shandong Normal University, Jinan, China

**Keywords:** leaf size, cell cycle, cyclins, MYB transcription factor, Chinese cabbage

## Abstract

**Introduction:**

The leaf, the main product organ, is an essential factor in determining the Chinese cabbage growth, yield and quality.

**Methods:**

To explore the regulatory mechanism of leaf size development of Chinese cabbage, we investigated the leaf size difference between two high-generation inbred lines of Chinese cabbage, Y2 (large leaf) and Y7 (small leaf). Furtherly, the transcriptome and cis-acting elements analyses were conducted.

**Results and Discussion:**

According to our results, Y2 exhibited a higher growth rate than Y7 during the whole growth stage. In addition, the significant higher leaf number was observed in Y2 than in Y7. There was no significant difference in the number of epidermal cells and guard cells per square millimeter between Y2 and Y7 leaves. It indicated that cell numbers caused the difference in leaf size. The measurement of phytohormone content confirmed that GA1 and GA3 mainly play essential roles in the early stage of leaf growth, and IPA and ABA were in the whole leaf growth period in regulating the cell proliferation difference between Y2 and Y7. Transcriptome analysis revealed that cyclins BraA09g010980.3C (CYCB) and BraA10g027420.3C (CYCD) were mainly responsible for the leaf size difference between Y2 and Y7 Chinese cabbage. Further, we revealed that the transcription factors BraA09gMYB47 and BraA06gMYB88 played critical roles in the difference of leaf size between Y2 and Y7 through the regulation of cell proliferation.

**Conclusion:**

This observation not only offers essential insights into understanding the regulation mechanism of leaf development, also provides a promising breeding strategy to improve Chinese cabbage yield.

## 1 Introduction

Leaves are essential to plant organs because of their roles in photosynthesis, respiration, photo-perception and transpiration (Tsukaya, 2002). Leaves provide plant growth and development energy through photosynthesis and respiration and store organic matter and mineral nutrients. Leaves arise from the shoot apical meristem (Scanlon, 2000), and the founder cells expand into a young leaf primordium in this stage (Poethig, 1997). In a previous study, the leaf outgrowth is determined by the cell division, which produces a certain number of cells with dense cytoplasm and the cell expansion, which make a specific cell size by cytoplasmic growth (Kalve et al., 2014). Cell division and cell expansion are complementary (Tsukaya, 2002; Beemster et al., 2003). Cell growth must be balanced by cell division and expansion for stable tissue growth and ideal leaf morphology (Sablowski and Dornelas, 2013). Thus, exploring the regulatory mechanism of leaf size can enrich the theoretical basis of leaf development and provide a basis for leaf size regulation and breed improvement.

The process of leaf development is a complex process regulated by genetic, environmental and plant hormonal factors, among which genetic factors are the intrinsic factors and play essential roles in leaf development (Tsukaya, 2003). The coordination of the two processes, cell division and expansion, is the basis for the final leaf size. The duration changes of either of these two processes can affect the leaf’s final size. In previous reports, *cyclin* (*CYC*) genes are involved in cell division and expansion, called the endoreduplication cycle (Xie et al., 2010). The combinatorial interactions between different CYCs and CDKs promote cell cycle phases (Komaki and Sugimoto, 2012). CYCDs in Arabidopsis primarily bind to CDKA to drive the G1 to S transition, while the CYCA2 combine with CDKBs to promote the G2 to M transition (Boudolf et al., 2009; Van Leene et al., 2010). It has been confirmed that the expression of *CYCD*s necessarily correlates with the presence of mitogens (De Veylder, 2019). Therefore, *MYBs* could activate *CYCAs* and *CYCBs* to promote the cell cycle in tobacco (Takatsuka et al., 2021).

Multiple genes control the plant cell division, and this regulatory network mainly includes the ANT (AINTEGUMENTA) pathway and TCP-GRF (TEOSINTE BRANCHED/CYCLOIDEA/PCF-GROWTH REGULATION FACTOR) pathway (Powell and Lenhard, 2012; Czesnick and Lenhard, 2015). Previous studies showed that in the ANT pathway, the *ARGOS* (*AUXIN-REGULATED GENE INVOLVED IN ORGAN SIZE*) overexpression promoted the cell division, resulting in larger organs, and downstream the *AXR1 (auxin-resistant 1)* to mediate cell division (Hu et al., 2003; Wang et al., 2010). As a downstream gene of *ARGOS*, *ANT* allows a more extended growth period of plant leaves by positively regulating the expression of *CYCD3*, increasing the size of plant leaves and floral organs (Mizukami and Fischer, 2000). Furthermore, ectopic expression of a *BrANT* increased the organ size and stomatal density of Arabidopsis (Ding et al., 2018). In the TCP-GRF pathway, *TCP* regulated by *miR319* negatively regulates the leaf organ size, and the overexpression of *miR319* caused a down-regulated expression of *TCP* (Class II), leading to excessive proliferation extended to the margins (Nag et al., 2009; Bresso et al., 2018). Moreover, the positive regulation of *TCP* (Class I) on cell proliferation has been identified (Li et al., 2005). In Arabidopsis, nine *GRFs* can regulate cell proliferation to promote leaf growth, *AtGRF1* and *AtGRF2* overexpression increase the leaf size, while the grf1/2/3 mutant showed a small leaf size (Kim et al., 2003). In addition, *BrGRF8* overexpression of Arabidopsis increased the leaf size by regulating cell proliferation (Wang et al., 2014). It has been reported that GRF regulates cell proliferation by the combination of GIF/AN3 (Horiguchi et al., 2005; Yuan et al., 2017). Moreover, the *miR396* also regulated the expression of *GRF* (Rodriguez et al., 2010).

Cell expansion is the second phase of leaf development: cell growth without division (Donnelly et al., 1999). An increase in cell size induced by cell expansion is essential to the complete growth and development of plant leaves (Breuer et al., 2010). Cytoplasmic accumulation and cell wall loose are vital factors affecting cell expansion (Czesnick and Lenhard, 2015). It has been reported that the expansion protein (EXP), xyloglucan endotransgluco sylase/hydrolase (XTH) and glycoside hydrolase (GH) are involved in the loose of the cell wall. The repression or overexpression of these related genes leads to changes in organ size (Goh et al., 2014; Lee et al., 2018; Hrmova et al., 2022). For example, the overexpression of *AtEXP10* promoted the increase of the cell volume and organ size, while the contrary result was observed when *AtEXP10* was repressed (Cho and Cosgrove, 2000). In addition, the disruption of *AtRPS13A (ARABIDOPSIS MUTANT OF CYTOPLASMIC RIBOSOMAL PROTEIN S13*) showed many enlarged cells and intercellular spaces in leaf blade (Ito et al., 2001). In contrary, the loss of proteases activity could inhibit the cell proliferation but not the cell volume, eventually led to the enlargement of leaves, flowers, seeds and other organs (Kurepa et al., 2009).Chinese cabbage is a typical leafy vegetable widely grown in Southeast Asian countries such as China, Japan and South Korea. Leaf size is a crucial trait affecting Chinese cabbage yield. So far, the molecular regulatory mechanisms of Chinese cabbage leaf size development remain unknown. A complete understanding of Chinese cabbage leaf development is critical to increasing the yield and editing the leaf shape of Chinese cabbage through genetic manipulation. In this study, two high-generation inbred lines of Chinese cabbage with significant differences in leaf size were used as materials. The molecular regulation mechanism that caused the leaf size difference was systematically explained through physiological, biochemical and transcriptomic analysis. This study provides help for the next step to improve the yield of Chinese cabbage by molecular breeding.

## 2 Materials and methods

### 2.1 Plant materials and physiological indexes

**Figure 1:**
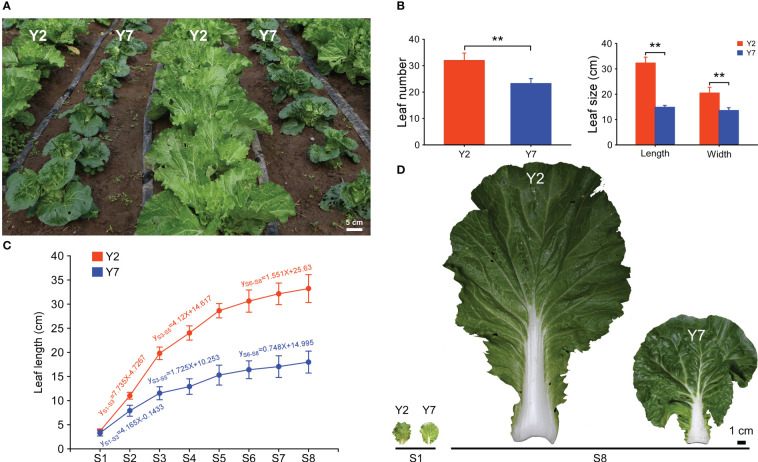
The growth of high-generation inbred lines Y2 and Y7. **(A)** Phenotypes of Y2 and Y7 in the field. **(B)** Statistics of leaf number and size 50 days after sowing. **(C)** Leaf length and growth rate of Y2 and Y7 from S1-S8. **(D)** The leaf of Y2 and Y7 at S1 and S8. * showed significant difference (Student’s t-test, **P < 0.01).

In this study, two high-generation inbred lines of Chinese cabbage, the large leaf size Y2 and the small leaf size Y7 were provided by our experiment field on August 20, 2020. We selected and marked the leaves with the same length (3 cm) from Y2 and Y7 on the 35th day after sowing. The length of ten marked leaves was measured every four days eight times, which were recorded as S1-S8. Meantime, others marked leaves were selected and cut from 1.5 cm at the top of the petiole for sampling (**Figure 1D**). Finally, 3-5 leaves were mixed as one biological replicate.

To verify RNA-Seq results, we prepared the seedling for the RT-qPCR. Seeds of “Y2” were germinated in plastic pots (25 × 25 cm) containing soil and vermiculite at a volume ratio of 1:1 and grown in our experiment field. One-month-old seedlings with 5-8 leaves were selected, and the leaves were selected and numbered in the order L1-L8 from new to old leaves. Three leaves cut from 1.5 cm at the top of the petiole were mixed for sampling.

All the samples were frozen in liquid nitrogen immediately and stored at -80 °C for further research.

### 2.2 Scanning electron microscope observation and cell number statistics

The leaves of Y2 and Y7 in S8 were collected for scanning electron microscope (SEM) observation and cell number statistics. The part near the middle of the leaves was cut into 0.5 cm×0.5 cm of square pieces and adhered to the glutaraldehyde fixation solution (25% glutaraldehyde 1 ml, 0.2 M pH 7.4 phosphate buffer 5 ml, distilled water 4 ml) for more than 12 h. First, the adhered samples were washed with phosphate-buffered saline (PBS) 5 times for 20 minutes each time. Then, the samples were fixed in 1% osmic acid for 2 h and washed 3 times using PBS, 20 min each time. After that, the samples were dehydrated by gradient with 50%, 70%, 80%, 90% and absolute ethanol, 20 min each time. Finally, the samples after ethanol dehydration were subjected to carbon dioxide critical point drying and then observed and photographed by scanning electron microscope (HITACHI TM3030, Japan).

According to the statistical rule, each intact cell and stoma was counted as one, and those with fewer than one cell or stoma near the edge of the visual field were always counted as 0.5. Y2 and Y7 each had three replicates.

### 2.3 The measurement of plant hormones

The freeze leaves of Y2 and Y7 cut in S1, S4 and S8 were ground to power with liquid nitrogen. First, 1.0 g power was added into a glass test tube and 10 ml isopropanol/hydrochloric acid buffer, and 8 µL 1 µg/mL internal standards, vibrating at 4 °C for 30 min. Then, we added 20 ml methylene chloride and vibrated at 4 °C for 30 min. The mixture was centrifuged at 4 °C, 13000 rpm for 5 min. The lower phase was taken and blown to dry with nitrogen in the dark. The samples were dissolved in 400 µL methanol (0.1% formic acid) and filtered with 0.22 µm film to measure IAA, IBA, IPA, ZT, TZR, GA1, GA3, GA4, GA7 and ABA. Plant hormones’ content was measured using HPLC-MS/MS (HPLC, Aglient1290; MS/MS: SCIEX-6500Qtrap). The standard curve was used to calculate the content. Three replicates of each assay were performed.

### 2.4 Transcriptome analysis

According to the manual, the S1-S8 leaves of Y2 and Y7 were collected for total RNA extraction using TRIzol reagent (Invitrogen, Carlsbad, CA, USA). Three replicates for each sample were analysed. Barcoded cDNA libraries were constructed using NEBNext^®^ Ultra™ RNA Library Prep Kit for Illumina^®^ (NEB, USA), and the 150bp paired-end reads were sequenced on NovaSeq 6000 platforms (Illumina). After the raw data were filtered, the sequencing error rate and the GC content distribution were checked, clean reads were obtained for subsequent analysis, and the mapped data were obtained by sequence alignment with the Chinese cabbage reference genome (Brara_Chiifu_V3.0, http://brassicadb.cn) using HISAT2. Use featureCounts to calculate the gene alignment. Fragments per kilobase of the transcript per million fragments mapped (FPKM) values were used to indicate transcript or gene expression levels. Principal component analysis (PCA) was conducted to evaluate the variation degree among the samples and groups. Weighted gene co-expression network analysis (WGCNA; PCC ≥ 0.8, minModuleSize = 30, cutHeight = 0.25) was performed on the transcriptome data to obtain co-expression gene modules and identify the network of genes regulating the leaf size of Chinese cabbage. The structural genes and transcription factors were organized into a connection network using Cytoscape software (Cytoscape 3.4.0). TBtool software was used to make the heatmap of gene expression. The RNA-seq data have been deposited in the NCBI Sequence Read Archive (NCBIvSRA) under accession number PRJNA895601.

### 2.5 RT-qPCR analysis

The total RNA of Y2 and Y7 leaves was extracted and used as a template, and a Takara Kit (PrimeScript 1st strand cDNA Synthesis Kit) was used to reverse-transcribe RNA into cDNA. Reactions were carried out using SYBR Green I Mix (QIAGEN) and ABI real-time quantitative PCR system. The analysis of each sample was repeated three times, and the 2^-ΔCt^ method was used for quantitative data analysis. The Actin gene of Chinese cabbage (BraActin) was used as an internal reference gene. In this study, all the primers (Qingdao WeiLai Biotechnology Co., Ltd.) are shown in “**Supplemental File 1**”. Three biological replicates were performed in all the experiments in this study.

### 2.6 Cis-elements analysis

DNA sequences of 2000 bp in upstream regions of the start codon (“ATG”) of cyclin genes were obtained from BRAD (http://brassicadb.cn) as promoters. PlantCARE (http://bioinformatics.psb.ugent.be/webtools/plantcare/html/) was used to predict cis-elements in promoter regions of cyclin genes (**Supplemental Files 2, 3**).

### 2.7 Statistical analysis

Three biological replicates were performed in all the experiments in this study. Statistical significance (Student’s t-test) and Pearson correlation coefficients were analyzed by using SPSS v24.0 software (SPSS Inc., Chicago, IL, USA), and a difference was considered to be statistically significant when P < 0.05 or P < 0.01.

## 3 Results

### 3.1 The leaf phenotype of Y2 and Y7

The growth of high-generation inbred lines Y2 and Y7 is shown in **Figure 1A**. 50 days after sowing, the leaves more significant than 2 cm in length of Y2 were more than those of Y7 (**Figure 1B**). Furtherly, the size of the giant leaf of Y2, including length and width, was significantly larger than Y7 (**Figure 1B**). For the growth trend of Y2 and Y7, the leaves (length of 3 cm) were marked 35 days after sowing, and the length of marked leaves was measured every four days for a total of eight times. As shown in **Figure 1C**, the elongation of leaves in Y2 and Y7 decreased gradually from S1 to S8, and a high growth rate was shown in Y2 leaves. During the growth of leaves, three phases of leaf elongation were identified, both in Y2 and Y7, S1-S3, S3-S5 and S5-S8. In these three stages, the proportion of Y2 and Y7 leaves elongation to the final leaf length was 48.94% and 46.6% (S1-S3), 25.9% and 18.9% (S3-S5), 14.8% and 13.4% (S5-S8), respectively. It indicated that S1-S3 was the critical period of determining the leaf size, followed by S3-S5, while S5-S8 showed the weakest effect.

**Figure 2:**
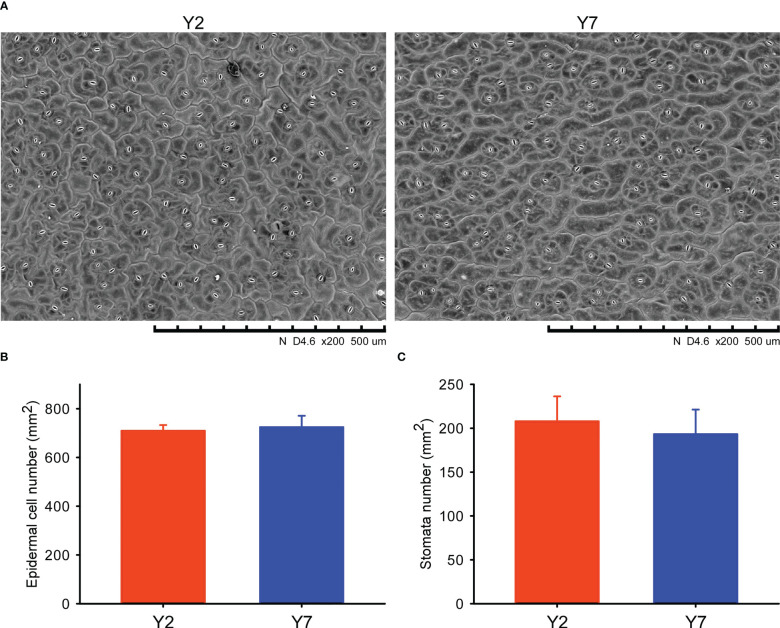
Scanning electron microscope (SEM) and statistical analyses of the Y2 and Y7 leaves. **(A)** SEM of Y2 and Y7 leaves. **(B)** Epidermal number per mm^2^ of Y2 and Y7 leaves. **(C)** Stomata number per mm^2^ of Y2 and Y7 leaves.

The SEM of the epidermal cell at S8 of Y2 and Y7 leaves was conducted (**Figure 2A**). The Epidermal number per mm^2^ and the stomata number per mm^2^ did not significantly differ between Y2 and Y7 (**Figures 2B, C**), indicating the same size of leaf epidermal cells. Based on the above, the difference in Y2 and Y7 leaf size was explained by the more vigorous cell proliferation of Y2 than Y7.

### 3.2 Differential accumulation of phytohormones between Y2 and Y7

**Figure 3:**
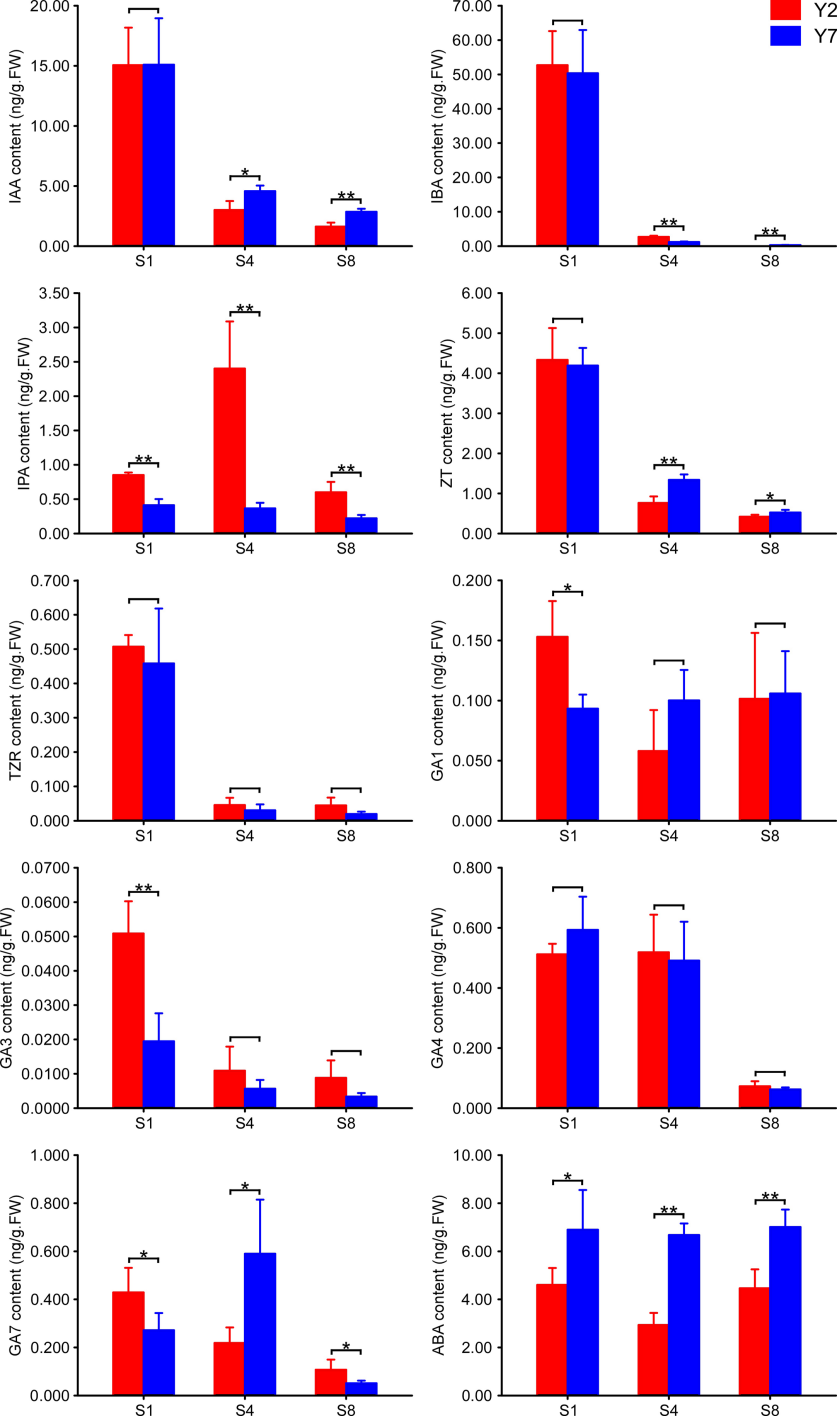
The content of plant hormones in Y2 and Y7 leaves at S1, S4 and S8. * showed significant difference (Student’s t-test, *P < 0.05; **P < 0.01).

The contents of auxins (IAA, IBA), cytokinins (IPA, ZT, TZR), gibberellins (GA1, GA3, GA4, GA7) and abscisic acid (ABA) in Y2 and Y7 at S1, S4 and S8 period were determined by GC-MS (**Figure 3**). A significantly higher IPA and lower ABA content were observed in Y2 at S1, S4 and S8. The higher IAA, IBA, ZT, TZR, GA1, GA3, GA4 and GA7 content were observed at S1 both in Y2 and Y7, indicating their vital roles in promoting the enlargement of leaf at S1. Even though, The IAA and ZT contents between Y2 and Y7 in S1 were no statistical difference, significantly lower contents in Y2 at S4 and S8 were observed. The content of IBA was similar at S1 in Y2 and Y7 and decreased as they grew. S4 showed a significantly higher IBA content in Y2, while S8 showed the opposite result.

### 3.3 Quality assessment of RNA-sequencing data

**Figure 4:**
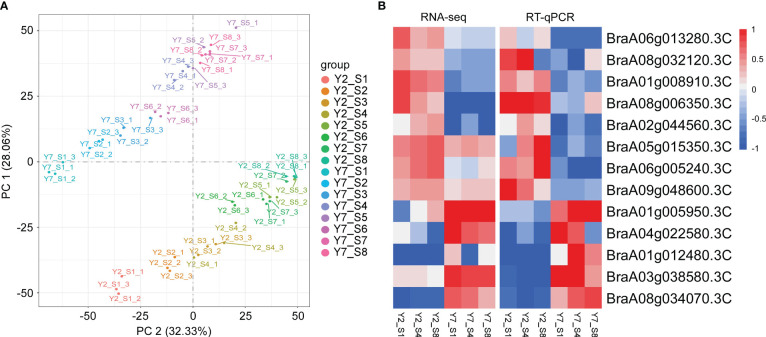
Transcriptome analysis of Y2 and Y7 from S1 to S8. **(A)** Principal component analysis (PCA) of RNA-Seq results. **(B)** The RNA-Seq and RT-qPCR results of genes were selected randomly. Each row represents the stage of Y2 and Y7. The red color indicates a positive correlation between the cluster and the sample. The blue color indicates a negative correlation.

To study the molecular regulatory mechanisms of the leaf size difference between Y2 and Y7, the transcriptome analysis (RNA-Seq) was conducted on Y2 and Y7 leaves from S1 to S8. The 48 transcriptome samples produced 2127.34 million clean data, more than 44.34 million per sample, with a percentage of Q20 and Q30 bases above 96.35 and 90.59%, respectively. Subsequently, all clean reads were compared and mapped to the Chinese cabbage reference genome sequence (http://www.brassicadb.cn/) by HISAT2 software. Transcriptional abundances were estimated using the fragments per kilobase of exon per million mapped reads (FRKM). The PCA score plot of FPKM showed that Y2 exhibited an apparent separation from Y7 in different stages, and three biological replicates of each stage were compactly gathered together (**Figure 4A**), indicating that the experiment was reproducible and reliable. This comparison indicated significant differences between Y2 and Y7 (p ≤ 0.05). To verify the RNA-Seq results, we selected 13 genes with significant expression differences between Y2 and Y7. Then, the RT-qPCR investigated the expression patterns of these genes. The relative expression of these 13 genes agreed with the RNA-seq results, indicating consistency in the RNA-seq data and the qRT-PCR results (**Figure 4B**).

### 3.4 The identification of cyclins regulated the cell proliferation difference between Y2 and Y7

**Figure 5:**
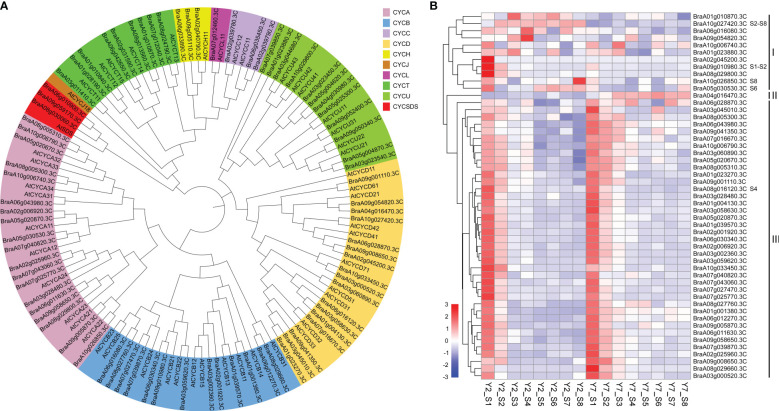
Phylogenetic analysis and expression patterns of the cyclins. **(A)** Phylogenetic tree of cyclin family proteins from Arabidopsis thaliana (At) and Chinese cabbage (Bra). The phylogenetic tree is generated by MEGA X software, according to the genetic distance model (Neighbor-joining tree). The same color in the phylogenetic tree indicates the same branch. **(B)** Heatmap representation of expression patterns in Y2 and Y7 from S1 to S8.

As mentioned above, the difference in leaf size between Y2 and Y7 is mainly due to cell division. Cyclins have been reported to involve to plant growth and development, especially Cyclin A, Cyclin B, and cyclin D play essential roles in the regulation of cell proliferation. Eighty cyclins belonging to ten subfamilies (A, B, C, D, H, L, T, U, J and SDS) were identified in Chinese cabbage. Subsequently, the phylogenetic tree of cyclins family proteins from Arabidopsis thaliana (At) and Chinese cabbage (Bra) was contrasted (**Figure 5A**), and 51 cyclins of Chinese cabbage belonging to A, B, and D classes were identified. Cyclin A, Cyclin B, and cyclin D of Chinese cabbage were mapped by the heatmap and clustered as their expression pattern. These cyclins were divided into three groups based on their accumulation in Y2 and Y7 at different developmental stages (**Figure 5B**). Cyclins in group I exhibited a high expression level in Y2 at S1 to S8, while group II showed a high expression level in Y7. The cyclins in group III expressed highly at the earlier stage of leaf size development, namely, the stage of S1 and S2, then decreased gradually in Y2 and Y7 (**Figure 5B**). It indicates that the cyclins in group I were mainly responsible for the difference in leaf size between Y2 and Y7, and group III mainly maintained the primary state of leaf growth. Furtherly, based on the expression patterns of 51 cyclins of Chinese cabbage, the *BraA10g027420.3C* (group I)*, BraA09g010980.3C* (group I), *BraA10g026850.3C* (group I), *BraA05g0030530.3C* (group I) and *BraA08g016120.3C* (group II) showed significantly high expression levels in Y2 than in that Y7, at S2-S8, S1-S2, S8, S6 and S4, respectively. Each of the five cyclins plays essential roles in different stages to regulate the leaf size difference between Y2 and Y7. According to Y2 and Y7, Chinese cabbage’s growth rate, Y2 grew faster than Y7 throughout the growth period. S1-S3 showed the highest growth rate than other periods in Y2 and Y7, and S1-S3 were the critical period leading to the leaf size difference between Y2 and Y7. Fortunately, we found that the expression of *BraA09g010980.3C* was significantly higher in Y2 than in Y7 at S1-S2. Meantime, the expression of *BraA10g027420.3C* at S2-S8 showed significantly higher expression in Y2 than in Y7. *BraA10g027420.3C* and *BraA09g010980.3C* were the critical cyclin genes to regulate leaf size development.

### 3.5 Identification of critical transcription factors regulating leaf size of Chinese cabbage

**Figure 6:**
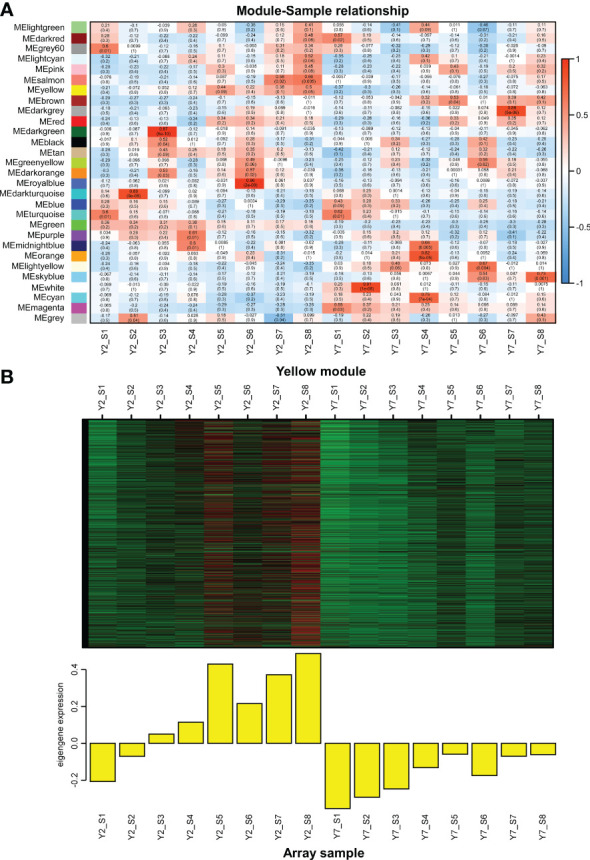
WGCNA of RNA-Seq data. **(A)** Module-Sample relationship. **(B)** The expression pattern of genes in the yellow module. The Pearson correlation coefficient (upper values) and the p-value (lower values) are indicated in the grid where each module and trait intersect. Each column corresponds to a module indicated by different colors. Each row represents the stage of Y2 and Y7. The red color indicates a positive correlation between the cluster and the sample. The blue color indicates a negative correlation.

Weighted gene co-expression network analysis (WGCNA) was used to identify the clusters of highly correlated genes. To construct the regulation network between *BraA10g027420.3C* and *BraA09g010980.3C* and their regulators, the correlation between gene matrix of different modules and different stages of Y2 and Y7 was analyzed by WGCNA and the correlation and corresponding p-values were presented in a digital form at the intersection of modules and samples. As a result, 25406 differentially expressed genes (DEGs) between Y2 and Y7 were identified and grouped into 29 modules based on their expression patterns (**Figure 6A**). Interestingly, we found that the *BraA10g027420.3C* was in the “yellow”, which was one module only positively correlated to the Y2 of S3-S8 (**Figure 6B**). On the other hand, *BraA09g010980.3C* was responsible for the leaf growth S1-S2 not located in any module.

**Figure 7:**
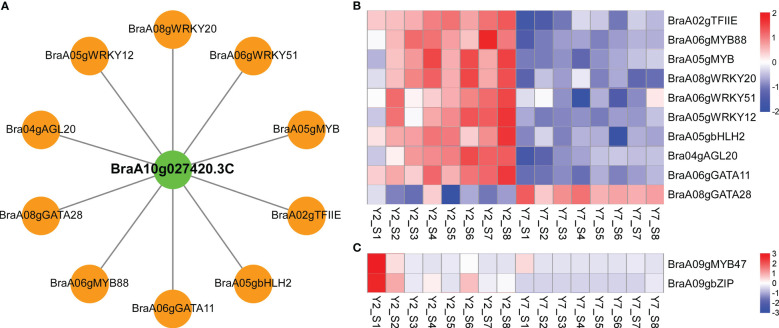
Co-expression of two cyclin genes and transcription factors involved the leaf development. **(A)** Connection network among top ten transcription factors and *BraA10g027420.3C* in Chinese cabbage. Green circles represent structural cyclin; orange circles represent transcription factors. **(B)** Transcriptome analysis of transcription factors co-expressed to *BraA10g027420.3C*. **(C)** Transcriptome analysis of transcription factors co-expressed to *BraA09g010980.3C*.

Furtherly, to explore the critical transcription factors regulating the two structural cyclins genes and their regulatory roles, we identified transcription factors correlated to *BraA10g027420.3C* and *BraA09g010980.3C* based on the module analysis and expression pattern, respectively. Firstly, the regulator network between *BraA10g027420.3C* and correlated transcription factors was conducted by Cytoscape software. According to the weight value, the top ten transcription factors correlated to the *BraA10g027420.3C* were identified, including *BraA02gTFIIE*, *BraA06gMYB88*, *BraA05gMYB*, *BraA08gWRKY20*, *BraA06gWRKY51*, *BraA05gWRKY12*, *BraA05gbHLH2* (BASIC HELIX-LOOP-HELIX 2), *BraA04gAGL20* (AGAMOUS-LIKE 20), *BraA06gGATA11*, and *BraA08gGATA28* (**Figure 7A**). Based on the RNA-Seq data, except for *BraA08gGATA28*, the other nine transcription factors showed high expression levels in Y2 but low in Y7, which was in line with the structural gene of *BraA10g027420.3C* (**Figure 7B**). In addition, two transcription factors with the same expression pattern as *BraA09g010980.3C* were identified. The expression levels of *BraA09gMYB47* and *BraA09gbZIP* (BASIC REGION/LEUCINE ZIPPER MOTIF) at the S1 and S2 were significantly higher in Y2 than in Y7, while there was no statistic difference at other stages between Y2 and Y7 (**Figure 7C**).

### 3.6 Expression pattern and cis-elements analysis

**Figure 8:**
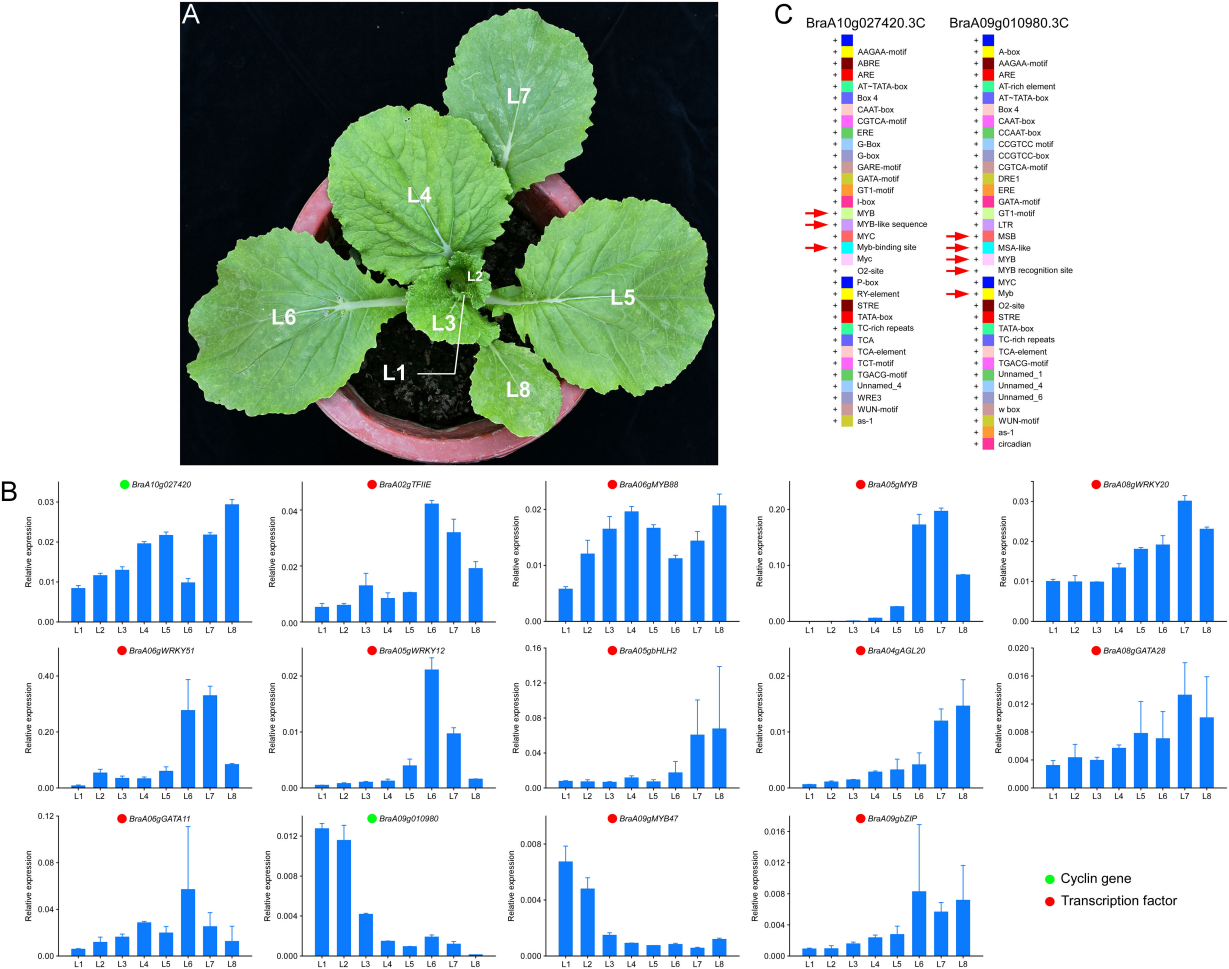
Validation of expression pattern and cis-elements analysis. **(A)** leaves positions of Y2. L1 is the youngest leaf, and L8 is the oldest leaf. **(B)** RT-qPCR validation of essential genes at different leaf positions of Y2. Green and cycles represent structural genes and transcription factors, respectively. **(C)** the cis-elements analysis of *BraA10g027420.3C* and *BraA09g010980.3C*.

To verify our results, RT-qPCR was used, and samples used for RT-qPCR verification were the leaves in different positions (L1-L8) of Y2 (**Figure 8A**). The expression of two cyclins, *BraA10g027420.3C* and *BraA09g010980.3C* and related transcription factors were confirmed (**Figure 8B**). For *BraA10g027420.3C*, the RT-qPCR analysis revealed the same expression trends as *BraA06gMYB88*. The expression pattern of *BraA09g010980.3C* was identical to the *BraA09gMYB47*. Subsequently, to confirm the regulation of MYB on *BraA10g027420.3C* and *BraA09g010980.3C*, we conducted the cis-elements analysis of these two essential cyclins. Interestingly, three and four MYB cis-elements were identified on the cyclin of *BraA10g027420.3C* and *BraA09g010980.3C*, respectively. The detail of these cis-elements is shown in **Figure 8C**. The cis-elements in the promoter of *BraA10g027420.3C* were MYB, MYB-like sequence and Myb-binding site, and the cis-elements in the promoter of *BraA10g027420.3C* were MBS, MAS-like, MYB, MYB recognition site and Myb (**Figure 8C**). These results confirmed the reliability of our above results.

## 4 Discussion

Leaf size is one of the essential traits crucial to the biomass of leaves and therefore affects Chinese cabbage yield. Therefore, leaf development has attracted the attention of botanists and biologists. The leaf development occurs from the formation of leaf primordia, followed by a period of rapid cell proliferation, and is directly related to the increase in cell number. Gradually cell proliferation becomes restricted until it stops proliferating, and then it begins to expand, with a dramatic increase in cell volume (Donnelly et al., 1999; Johnson and Lenhard, 2011). In our study, the cell division is responsible for the leaf size difference between Y2 (large leaf size) and Y7 (small leaf size). In a previous report, the leaf size difference is mainly due to the difference of cell number caused by the prolonged cell proliferation period (Powell and Lenhard, 2012). Therefore, consistent with our SEM results, the difference between Y2 and Y7 was probably caused by cell number (**Figure 2A**).

Phytohormones, as mobile signals, are essential regulators of leaf size by regulating cell differentiation and expansion. Cytokinins (CKs) are well-known for controlling the cell cycle (Rath et al., 2022). In our research, the IPA was the critical hormone regulating leaf size throughout the growth stage. Cytokinins regulate many cell cycle genes, such as B-type cyclins (*CYCB*), D-type cyclins (*CYCD*) and cell division cycle 2 (*cdc2*) (Kuluev et al., 2018). The CKs promote the growth of plant organs by stimulating cell proliferation, with CKs depletion or overproduction resulting in smaller or larger leaves (Werner et al., 2001; Bartrina et al., 2011). CKs and auxin interact with each in the cell cycle and leaf expansion providing positive feedback regulation of leaf growth (Davies, 2010). The crucial roles of GA1 and GA3 in regulating an earlier stage of leaf growth were confirmed in our study. GAs induced the *SAUR* (*SMALL AUXIN UP RNAs*) expression, and *SAUR* promoted cell expansion by activating plasma membrane H+-ATPase (Ren and Gray, 2015; Nagpal et al., 2022). The previous study also reported that GA could regulate an overlapping set of *SAUR* to maintain cell elongation by constructing the DELLA-ARF6-BZR1-PIF4 complex (Oh et al., 2014; van Mourik et al., 2017). It has been reported that the IAA and GA can activate the cell wall structural proteins and enzymes, such as EXP and XTH, to induce the loose of the cell wall and then the cell elongation (Kou et al., 2021). The auxin promotes organ growth by stimulating the expression of *ARGOS*, which prolongs the expression of *CYCD3* and *ANT* (Mizukami and Fischer, 2000; Hu et al., 2003). *EBP1* (ErbB-3 epidermal growth factor binding protein) could respond to auxin signals and stimulates the expression of *CyclinD3;1* (Horváth et al., 2006; Powell and Lenhard, 2012). The S1 showed a higher auxin level than S4 and S8 in Y2 and Y7, but there was no significant difference between Y2 and Y7 (**Figure 3**). This result indicated that the auxin should be mainly involved in the basic growth and development rather than the critical factor determining the size difference between Y2 and Y7 leaves. This coincides with reports that auxin mainly modulates cell and leaf expansion (Davies, 2010). ABA was negatively regulating the leaf size in the whole leaf growth stage. As reported in previous studies, the molecular basis of the antagonistic relationship between CK and ABA was unraveled. *SnRK* (Sucrose nonfermenting1-related kinases), as the essential positive regulators of the ABA signaling pathway, directly interacts with *ARR5* (phosphorylate type-A response regulator 5), a negative regulator of cytokinin signaling (Huang et al., 2018). Our study also demonstrated that GAs, CKs and ABA are essential in regulating the leaf size difference between Y2 and Y7. GA1 and GA3 probably play a significant role in the early stage of leaf growth, while GA and ABA act at S1-S8.

The cyclin genes are the primary regulator of plant cell cycle progression, which cooperated with cyclin-dependent kinases (Qi and Zhang, 2019). There are ten classes of cyclins in Arabidopsis consisting of approximately 49 cyclin genes has been reported, and only the classes of A, B and D were well known to regulate the cell cycle (Wang et al., 2004). The D-type is considered the regulator of the G1-to-S transition, the A-type plays a vital role in controlling the S-to-M phase, and B-type is mainly responsible for controlling the G2-to-M transition (Qi and Zhang, 2019). At different stages of the cell cycle, different CYC proteins bind to CDK kinase to form specific CYC-CDK complexes, which trigger the transition between G1/S and G2/M phases of cells, and control cell proliferation (Komaki and Sugimoto, 2012; Wood and Endicott, 2018). In our study, most cyclin genes showed a high expression level at S1 and S2, consistent with the highest growth vigorous proliferation of Y2 and Y7 at S1-S3. It indicated that most cyclins regulated the basic cell prefoliation of leaf in Y2 and Y7. Interestingly, we identified that the expression of *BraA09g010980.3C* (CYCB) does not belong to any module, significantly higher in Y2 than in Y7 at S1-S2, which probably the critical cyclin. In addition, *BraA10g027420.3C* (CYCD) was mainly responsible for the leaf growth at S3-S8 with a long growth duration of Y2.

Transcription factors regulate almost all major biological processes at the transcription level by binding to the cis-elements of target genes through the DNA-binding domain (DBD) (Velthuijs et al., 2021). Several transcription factors, such as *WRKY* (Yu et al., 2021), Zinc Finger of Arabidopsis thaliana (*ZAT*) (Fan et al., 2021), *MYB* (Okumura et al., 2021), *WUSCHEL-RELATED HOMEOBOX5* (*WOX5*) (Schwedersky et al., 2021), *bHLH* (Li et al., 2019) have been reported in the regulation of cell cycle, which was consistent to our study. Among these transcription factors, the expression pattern of MYBs were as consistent with the vital cyclin genes, *BraA10g027420.3C* and *BraA09g010980.3C*, thoroughly. Furtherly, the cis-elements analysis of these two cyclin genes confirmed our results that the MYB, MSA, Myb-binding site, MYB-like sequence, MBS, MAS-like, MYB recognition site and Myb cis-elements play essential roles in the regulation of cell cycle. As previously reported, *MYBs* play an important role in plant secondary metabolic regulation, hormone responses, cell differentiation and cycle regulation. In Arabidopsis, MYB3R binds to the promoters of the M phase-specific activator (MSA) of target genes to regulate the transcription of the G2/M phase-specific gene (Sumiya, 2021). In the G2/M phase, AtMYB3R1 and AtMYB3R4 active the CYCA2, CYCB1 and CYCB2 genes by recognizing and combing with MSA element (Nomoto et al., 2022). Furthermore, MYB3R1 and MYB3R4 positively regulate cytokinesis by activating *Knolle* transcription (Saito et al., 2015). In addition, *AtMYB88* encode closely related and atypical two-MYB-repeat proteins, which, when mutated, result in excess divisions of stomata in contact (Xie et al., 2010). Thus, we speculated that the two essential cyclins promoted the cell cycle under the regulation of *MYBs* transcription factors in Chinese cabbage.

## 5 Conclusions

In conclusion, this study conducted a comprehensive analysis of the difference in leaf size between Y2 and Y7, and the regulatory mechanism. We confirmed that GA1 and GA3 mainly play a role in the early stage of leaf growth, while IPA and ABA play a vital role in the whole growth period of leaves in regulating the cell proliferation difference between Y2 and Y7. In addition, two essential cyclin genes that are involved in the regulation of leaf size differences between Y2 and Y7 were identified in this study. Further studies showed that the transcription factor of MYBs plays a vital role in the transcription and expression of the above two critical cyclin genes. Taken together, this study not only provided clues to understanding the molecular mechanism of leaf size regulation in Chinese cabbage but also established a foundation for improving the yield of Chinese cabbage by molecular biological methods in the future.

## Data availability statement

The original contributions presented in the study are publicly available. This data can be found here: https://www.ncbi.nlm.nih.gov/bioproject/PRJNA895601.

## Author contributions

FW and JG supervised and conceived this project. LW wrote the paper. SZ, YeZ and FW revised the paper. LW, YeZ and SZ performed the formal analysis. LW carried out qRT-PCR. All authors contributed to the article and approved the submitted version. 

## Supplementary material

Supplemental File 1 RT-qPCR primer.

Supplemental File 2 and 3 Cis-elements in promoter regions of cyclin genes.

Supplemental File 4 RNA-Seq data.

Supplemental File 5 Metabolome data of plant hormones.
