# Supplementary material for: Integrative analysis of physiology, biochemistry and transcriptome reveals the mechanism of leaf size formation in Chinese cabbage (Brassica rapa L. ssp. pekinensis)
Source: Front Plant Sci. 2023 Apr 6;14:1183398. doi: 10.3389/fpls.2023.1183398 (PMC10118011; doi:10.3389/fpls.2023.1183398)
Supplement: Supplemental File 1 — RT-qPCR primer. [file DataSheet_1.pdf]

(+) sense strand
MSB
MSA-like
MYB
MYB recognition site
Myb

BraA09g10980.3C promoter Cis element analysis

TTAATTTTTTTTTAATACAATGCTAGTTTTTTATTTTAAAATTTCTATAGAATATAGAATGAACATGGATGAATCAACAAC

AAAGTGCTTGTTCAGACAAGGATCAACTAGTGTTTGGATTGAAAGTATTTGAATTTGATTTTTTAACCGGAAAAGAG

TTTAAAGACAATGACAATTAAGTTGAAGAGCATGAGAGGAAGCCTCATGTACTGGCAGGCGTGGATGAGACCGAC

GACTTGGCCTTTGAGGGAGTTTCTTTTGCCGGTTTGGTGAAGCTCCGCGAGTGTCCATCCTTTGGGAGCTAAGA

AACGGTCTTCGTTGAGGATAGCAAGTGCGTTTGCGAATAGCAGCAACCCTTCCATCAGTGTCCAGAATCCCATGA

TGATTTCTCGAGTAAATGAGGAAATTTGACTTGTAGTAGCTCCAAGCCTGGTTGGAATCCTTGGCCTAACTACAGC

TGAGTCGAATCGGGGTTAAAGGGTTAAAGTGTTTGTTTAATTAGAAACATGAATTGTATGTGTTAAAAAGGAAAGAA

CCCCATGGATTAGTATAGATGTGGTGCAATTGAATTTTAAGGAGAAGGCTGGGCTTCACAATGTATATGTGTGAAAT

CTCTATATGTGTAGTCCAGTCCCATCAATCTTTTTCCCAAGTCAACTCCAGCCTAATTATTTAAGTTTCGGTTGAGTT

TGAGAAATTTTTTACTTACCAAATCGCTTGATCAAGTCATCTGTAGACCGATCTCGTTCGCAAGCATGCATATATCA

GTAATGGCATGAGAATGAGATGGTCCCCTCATTGTCAGAACTCTTTGGCGTTTGGTTTACGAAAACAATGCCTTTA

AGACATTATTAATTAATTTGACAAAGATGATTAGGAGGCTCCCCCATGTTCACGGCTTCATTCCCTGGCATTTATGC

CTAGTTTTTCGTGAACATTGACTGTTCATAATTCGTATGCTTATGGTAAGTAAAATGATTACAATTCTTTGGTTCTCTG

MBS(+)/Myb(+)

TGCCATAAAATTTCAAAGTTGCAACTCTCGAATTCACAACTGATAATATAAACAGTTTAACTTGTAGGGTCTAGTTGA

TTTAAATGCAAAGGGTACAAATGCAGATCCATAAGTAAAAATCTGTGAAGTAAACACTTTTACGGTTTCAAGTGTTAT

TAACTTATTATATATATTTATAAAAAAATACTATACACATTCTTGTTATTTTCAAATCCACATTGTCTAGTTGAATCTATTG

CGAACATAGGGAATAGTTGATATTAGTTATATTAATTAACTTGGTATGAATCTTTGACCCATATTAAAAAACAAACTTG

ATATGAATGATATGATGTACTTTGAACAGAGAAGCTAAGTTTTAGATTGTTTTCTTTCCAAAAATATCTTAGGCTATTA

AAAATATTCAAGCAAAAGAATCACTAATTAAGAACTGTCACGGGGCAGCATAATTCTCGGTAGGGAATCTGGTTCT

GTCTTCTACTGAAATTAGTCTGAGATAGAGCATATGATTAGCTCTGTAAATACGGATATGAATAA AGCTAAATTACTG

MYB(+)

ATTATTTATGATAATAATTACTCCAAAAATTTAAAAGAAAATCAAACACTAGTTAGGCCGCGCCTATGGAGCGCAACC

MYB(+)

ATTTGGTCTGAATAAAAGTAGAAGCCAACCAATAAAAGAAAAGATTAATAAATAAAAGTTTGAAATAAGAAAAGACGA

AGCCACATGCCTCGGACCAAAGTTACCGTTAAGTCTTCGTGCCAGACTTGGAAGTAAATCGTTTTATTCTCATAA T

MSA-like(+)/MYB recognition site(+)

CCAACGGTCATATCTGATCCTAGCCGTCCATTTAAAAAATAAAATAAAAATTACACTTATTTCTATTTTGATTTATATTC

GTCAATTCATAGTCACTTGCCTATATCAACCCCTTGGTCAGATTCGACTGTATCAGACTATAAAGATTCTCTTTCGCT

TA AT G C T C T T C C T C TA G T C T C T C T G G T G G G T T T T C T C T C G G A A G A AT TA A A A C G A A C A A A A AT G
