# Supplementary material for: Integrative analysis of physiology, biochemistry and transcriptome reveals the mechanism of leaf size formation in Chinese cabbage (Brassica rapa L. ssp. pekinensis)
Source: Front Plant Sci. 2023 Apr 6;14:1183398. doi: 10.3389/fpls.2023.1183398 (PMC10118011; doi:10.3389/fpls.2023.1183398)
Supplement: Supplemental File 2 and 3 — Cis-elements in promoter regions of cyclin genes. [file DataSheet_2.pdf]

(+) sense strand
MYB
MYB-like sequence
Myb-binding site

BraA10g27420.3C promoter Cis element analysis

TCAGAGTTCTACTATGAGTTCATCATTCTACTTATACCAGAACTCCTAGCTCAGCATGTCCAAGGTCTTTAGTTAAAC

MYB(+)/Myb-binding site(+)

AACAGATTTTGTCATTGCCTGCTAGGACATTCTAAATTTTAATGCTTCATTCACCTTTAGGATTTTGAAGAAGACATA

GCTGAGCTTGGCACGCTGGGAAAAGAAAACCTACAAAGACGGCACTCTCGTTTTGCGGCTGCTTAGGGATCTTG

AGGAAGGGTTGGGGAGTGGTATCATACCACTGGTATGGAACATGTTTCCACCACCAGATTTCAATCTCAAGATGA

CCTTAACCTTATCTCAAGACTGAAAAAGTTATTGAATTTGTTTATGATGATTATGTGCACTGCTTGTTGATTTGTCTAC

TCTTATCAAAAAACGTCTAAAACTATGTTCTCTAAAATTTGTCATCTCTTGACTTTGCTCAAGCCTTGAAAACCTCTT

TGACTCTTCTACGATCTGCTCTGGTTCTAGTTTGAAACAAACAATCTAAGGTCTCCTTTAAAGTTAGAAACTAACAC

GCTAAGGTCTCCTCTGGTTCAAGTTAGAAACCAACAATCTTGGTCTCATTGTTTTT GGGGTGAATATAGTAATAATT

AACTTCTTTCTGTAAATATAATAAAACCAGTCAAAGTCCAAGCAACACAATTTATGTAGCATAGAGCGACCATAAGAC

GTGCACACACACATGCAACTATGCAAGATTCGAGGTGAGAATCAAAAGAAGTCGAATCTCATTGCACAAAGAGAT

GCAAAACATCAAAATTGTACTTGGACCACTTTGTCTGCATTGCATGCATGAAACGATATATTAAATAGAACACAGTCT

TTGGTCTATTTCTGCGACATACATAATTGTTTTCTCATATATTACTTCAACTTTTCATTTCTCCCACCAATCTTTTCAG

AGAAAGATACTGTAAACATTAAAAACTAAGAACCAAAGTTGCTCGCTCACACGTGATTCCAGTCCACCTCCACTAC

AAAGTTATACAAATTGTTGTCAAGTTGAAAGTTATATTTGATTAATAGATATAAAACCTTGAAATGAATACTTTTTAGCT

AGGGGCATCATTCTTTTGTAAGAAA CCCACCGATCTCTTTTCTTTTGTTAGGGTGTTCGCTTTACTTCCTTGTTTTT

TACTGCAAATAAACATTGTGATGCAGAGAGGACCGTAGCCAAAAAAAACCTTGAGATTAGACTAGAGTGTAATGTT

MYB(+)/MYB-like sequence(+) MYB(+)

ATAACCAAGTGTTCATACCTCAACCAAAAGGTATCAATCAAGACAAAGTTTAGAAGTCATAGAAACGAGCAAGTCG

AACAAAACTATTATTACATATGGAGCGAAACTAATATACACAACATGTTTTTGGGATTAGGAAGATTATATATGTGACG

ACCTAGAACCTTATGATAAAATAAATTATAGTGCAATGTTGTCTAGTCACATGACATGATGATAATCAGAGGTTGTGA

MYB(+)/MYB-like sequence(+)

AAAATGTGGAGAGGTCTGAGACGAATAGCTTTTGTAGTCCACTAACCAATAAAAGTTACAATTACAAGCCTTTTTCG

AAATATAGTGGTGAAAGAGAGATTCGAAGTTCATTTAATCTCAATGATTCACAATCTATGGGATTAAACATTCGCTGC

MYB(+)

ATCTTCAGATTATTTGAATTGTTATACATTTTGGTATTAAAAAATCTAAAAAATAAGAGCAACCATTATTATTTTTTCACA

TCCATTTTCCAAATTTAAAATAAAAATATTTGTTTCCATGCTAATTTGAATTTTCTCCATCACATTTCAATCTCTTTACT

CTAAAGCCACATCTTCATCTATATATCAATAATTTATCCTCTCTGTCTTTTGTTTTATCTCTTCTGACCCCCAAAAACA

ATCTCAAAGTAAAACAAACAAAGAAGAACAAAATAAAATAAAAACAGAGACAAAATCTCTTTTTCAGTTCATTATCTC

T TA ATA C TATA A A A A C G A A A A A A A A C T T G TA A A C A G AT C G G A A A A A G G C A A C C T C TATA AT G
