# Supplementary material for: Integrative analysis of physiology, biochemistry and transcriptome reveals the mechanism of leaf size formation in Chinese cabbage (Brassica rapa L. ssp. pekinensis)
Source: Front Plant Sci. 2023 Apr 6;14:1183398. doi: 10.3389/fpls.2023.1183398 (PMC10118011; doi:10.3389/fpls.2023.1183398)
Supplement: Supplemental File 4 — RNA-Seq data. [file Table_1.docx]

**Supplement Table 1.** The qRT-PCR Primer.

| qRT-PCR Primer | | | |
| --- | --- | --- | --- |
|  | ID | 5’-3’ | Sequence |
| 1 | BraActin | F | GGAGCTGAGAGATTCCGTTG |
|  |  | R | GAACCACCACTGAGGACGAT |
| 2 | BraA10g027420.3C | F | CCATCGGAGGAGGACGAAGA |
|  |  | R | ACCGAAGTTCCTCACATGCC |
| 3 | BraA09g010980.3C | F | TCGTTGCCTACGCAATACCC |
|  |  | R | GCTCGATCAAGAACGATGCCA |
| 4 | BraA09g057130.3C | F | GCTGTCCTCGGAAACAGGTG |
|  |  | R | AGTGATCGTGACGGTGAGGT |
| 5 | BraA09g011080.3C | F | CACCCACATCGCCAGCTAAA |
|  |  | R | GAGACGTGGTGAGTGGAGGA |
| 6 | BraA02g028520.3C | F | GAACACTGCGCAGAGGAGAG |
|  |  | R | CTCGGGAAGATGAGCGTTGG |
| 7 | BraA06g039600.3C | F | TTGTGCCCATGTCGGTTGAG |
|  |  | R | AGAGAACTGTGGGCTTGGGA |
| 8 | BraA05g035590.3C | F | GCTCGGGCTTCTCAAGTACG |
|  |  | R | TGCCGGTTGTGATGTCATGG |
| 9 | BraA08g019780.3C | F | TGGTGTTGGTATGGCTGCAC |
|  |  | R | GCAACGTCTCCACCATCGTT |
| 10 | BraA06g028240.3C | F | GACATTTACCGGCGAAAGCG |
|  |  | R | ATGGCCCATCTCCTTCGTCT |
| 11 | BraA05g004630.3C | F | CGATGGCTACAAGTGGCGTA |
|  |  | R | GAGGGAATGTGGCTGTGACG |
| 12 | BraA05g015350.3C | F | TGGCTGGCAAGCAGAATGAG |
|  |  | R | CCCTTTCCGCGATACTGTGG |
| 13 | BraA04g031640.3C | F | TCAGTGCTTTGTGATGCGGAA |
|  |  | R | ACCGGTTTGTTGCTGATTCG |
| 14 | BraA08g002520.3C | F | GCTGGGAGTCCAGTCAAACG |
|  |  | R | CGATTCCACAGTGCCGACAT |
| 15 | BraA06g005240.3C | F | GAACTCGAGCCTCCTCCCAT |
|  |  | R | CAGACAAGGCTGCAGAAGCA |
| 16 | BraA06g013280.3C | F | CAAGGACTCACGGTTTCGCA |
|  |  | R | TTCGTGGAGCTCTTCCGCTA |
| 17 | BraA08g032120.3C | F | CCGAGCGAGAGGTTAACACG |
|  |  | R | TCGCTTCTCCTTACGGCTCA |
| 18 | BraA01g008910.3C | F | GCAGCAACCGCAGATTGTTC |
|  |  | R | CTGACAGGTCGCAACTCCAC |
| 19 | BraA08g006350.3C | F | CAGCGGAAGAGCAGTGTTGT |
|  |  | R | CGGCCATGTTCCTGATAGCG |
| 20 | BraA02g044560.3C | F | GGCAATGGGATTTGCCTCCT |
|  |  | R | CTGCAAACAGTGGGCCTGAA |
| 21 | BraA08g034070.3C | F | GAAACAGCAGCAGCAGCAAC |
|  |  | R | CAGCTCCGTAACACCGCTTT |
| 22 | BraA09g048600.3C | F | TGCTAAGACCGTGACGCCTA |
|  |  | R | CTATGCGCTTAGCGTGTGGT |
| 23 | BraA01g005950.3C | F | GGTGGCTCTCCTTCGACTCT |
|  |  | R | ATCGGATGCCTCACAATCGC |
| 24 | BraA04g022580.3C | F | GCAACTGCCACTCAGCATCT |
|  |  | R | GCAAAGAGCCTAGCTGCACA |
| 25 | BraA01g012480.3C | F | TCCATGGCCAGCAACATTCC |
|  |  | R | TGGCTAATGTCGTCCATGCG |
| 26 | BraA03g038580.3C | F | GATGCAGCTGACAAGTGGCT |
|  |  | R | AGCTGCATGAGATGGTTGCC |
